# Application of monoclonal antibodies in quantifying fungal growth dynamics during aerobic spoilage of silage

**DOI:** 10.1111/1751-7915.13552

**Published:** 2020-03-10

**Authors:** Kate Le Cocq, Bethany Brown, Christopher J. Hodgson, Jamie McFadzean, Claire A. Horrocks, Michael R. F. Lee, David R. Davies

**Affiliations:** ^1^ Rothamsted Research North Wyke Okehampton Devon EX20 2SB UK; ^2^ Bristol Veterinary School University of Bristol Langford Somerset BS40 5DU UK; ^3^ Silage Solutions Ltd. Bwlch y Blaen Pontrhdygroes Ystrad Meurig Ceredigion SY25 6DP UK

## Abstract

Proliferation of filamentous fungi following ingress of oxygen to silage is an important cause of dry matter losses, resulting in significant waste. In addition, the production of mycotoxins by some filamentous fungi poses a risk to animal health through mycotoxicosis. Quantitative assessment of fungal growth in silage, through measurement of ergosterol content, colony‐forming units or temperature increase is limiting in representing fungal growth dynamics during aerobic spoilage due to being deficient in either representing fungal biomass or being able to identify specific genera. Here, we conducted a controlled environment aerobic exposure experiment to test the efficacy of a monoclonal antibody‐based enzyme‐linked immunosorbent assay (ELISA) to detect the proliferation of fungal biomass in six silage samples. We compared this to temperature which has been traditionally deployed in such experiments and on‐farm to detect aerobic deterioration. In addition, we quantified ergosterol, a second marker of fungal biomass. After 8 days post‐aerobic exposure, the ergosterol and ELISA methods indicated an increase in fungal biomass in one of the samples with a temperature increase observed after 16 days. A comparison of the methods with Pearson's correlation coefficient showed a positive association between temperature and ergosterol and both markers of fungal biomass. This work indicates that the technology has potential to be used as an indicator of microbial degradation in preserved forage. Consequently, if it developed as an on‐farm technique, this could inform forage management decisions made by farmers, with the goal of decreasing dry matter losses, improving resource and nutrient efficiency and reducing risks to animal health.

## Graphical abstract

Current techniques for detection of aerobic spoilage in silage include measurement of changes in temperature and ergosterol concentration. Here, we investigate a novel approach to detection of fungal proliferation in silage through application of a monoclonal antibody based method previously described for detection of fungi in soil and medical settings.

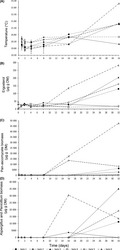

## Funding Information

This work was funded by the Society for Applied Microbiology Students into Work Grant and supported as part of Rothamsted Research's Institute Strategic Programme – Soil to Nutrition (BS/E/C/000I0320) funded by the UK Biotechnology and Biological Sciences Research Council.

## Introduction

The ensiling of forage is fundamental to the diets of ruminants and equids, particularly where climatic conditions require additional feed during winter months or where livestock are housed continuously within more intensive systems (ca. 8% of UK dairy herds; March *et al.*, 2014). In the UK, grass (predominately *Lolium* spp.) and maize (*Zea mays*) are commonly ensiled, with the production of 50 million tonnes of fresh matter (FM) annually (Wilkinson and Toivonen, 2003). Efficient ensiling of forage relies on a rapid decline in pH and an anaerobic environment being maintained after sealing of the clamp or bale, which reduces the breakdown of protein from plant enzymes and prevents growth of undesirable microorganisms (Bolsen *et al.*, 1996). Compromise of the anaerobic environment through ingress of oxygen enables aerobic microorganisms, particularly fungi (Auerbach, 1996) to begin respiration of readily available energy sources (i.e. water‐soluble carbohydrate and/or starch) within the ensiled forage. Aerobic spoilage of silage is estimated to be responsible for £110 million annual losses to the UK farming industry (Williams *et al.*, 1995). This can account for circa 10% dry matter (DM) losses on an individual farm (McDonald *et al.*, 1991). In addition, livestock, including horses, are also susceptible to disease caused by filamentous fungi (Cafarchia *et al.*, 2013; Seyedmousavi *et al.*, 2018). Usually, aerobic spoilage is initiated when the silage clamp is opened for feed‐out and is detectable by an increase in temperature of the silage near to the feed‐out face (Muck *et al.*, 1991; Muck and Pitt, 1994). However, very often there are regions of silage where oxygen ingress occurs during the storage process (Woolford, 1990). Several factors such as poor consolidation at filling, poor sealing and damage to the plastic by wildlife and machinery are considered key contributors to the risk of aerobic spoilage (Wilkinson and Davies, 2013).

Our current knowledge of microbial proliferation during aerobic deterioration is based on the sequence of events occurring in a well‐preserved silage that has been opened and exposed to oxygen. In this situation, the silage is of high preservation quality with a low pH and high levels of lactic acid (120–160 for clamp silages and 5–75 g kg DM for baled silages) (Coblentz and Akins, 2018; Kung Jr *et al.*, 2018). Current evidence suggests that yeasts (commonly of the genus; *Candida*, *Pichia*, *Geotrichum Rhodotorula* and *Hansenula*) initiate aerobic spoilage as they utilize lactic acid, converting it to carbon dioxide and water and thereby increasing silage pH (Jonsson, 1991; Pahlow *et al.*, 2003; Reboux *et al.*, 2006). The chemical changes induced by primary aerobic colonizers enable a microbial succession of facultatively anaerobic and then secondary aerobic microorganisms such as the *bacilli* and acetic acid bacteria, followed by tertiary aerobic colonizers such as filamentous fungi which then proliferate and further utilize energy sources reducing silage nutritive value (Lindgren *et al.*, 1985). In addition, filamentous fungi often produce spores and mycotoxins which have many negative effects on animal health leading to mycotoxicosis (Fink‐Gremmels, 2008) with further potential risks to humans (Zhao *et al.*, 2013). However, in most farm silos, there are regions where oxygen is likely to be presented for most of the storage process due to poor management factors highlighted earlier (Woolford, 1990). Under these conditions, it is likely that the microbial ecology is less defined in terms of primary and secondary colonization processes. Field borne pathogenic and saprophytic fungal species continue to grow in silage until the level of oxygen becomes too low, inhibiting their further growth. The conditions under which filamentous fungi produce mycotoxins in silage has not been clearly defined (Cheli *et al.*, 2013), and as such, the importance filamentous fungi play during the silage preservation process may have been underestimated (Fink‐Gremmels, 2008).

Aerobic spoilage of silage on‐farm is most commonly identified through changes in temperature and the appearance of mycelium or spores on the silage at feed‐out (Kung, 2011; Wilkinson and Davies, 2013). Although increase in temperature is a symptom of aerobic spoilage, there are factors that may affect the extent to which it is a reliable measure, for example the ambient temperature at harvest, the natural insulation properties of the forage or the rate of heat dissipation (Borreani and Tabacco, 2010). Therefore, in practice, this indirect method can be subjective and unreliable in determination of the extent of aerobic spoilage. Laboratory‐based methodologies used to assess fungal proliferation in environmental samples consist of enumeration of colony‐forming units (CFUs) by serial dilution and culture on a range of media (Auerbach *et al.*, 1998; Bueno *et al.*, 2004; O’Brien *et al.*, 2007; Keller *et al.*, 2013), molecular [quantitative polymerase chain reaction (PCR)] (Niessen, 2007; Nicolaisen *et al.*, 2009; Richard *et al.*, 2009; Muck, 2013) and biochemical (ergosterol) measurements, in which most work has focused on leaf litter and soils (Gessner *et al.*, 1991; Djajakirana *et al.*, 1996; Gessner, 2005). Ergosterol is a sterol found in the cell wall/membrane of fungi and can be extracted using cyclohexane and measured by high‐performance liquid chromatography (HPLC) (Rousk and Bååth, 2007). Ergosterol has previously been acknowledged as having a role as a marker of fungal load in a range of environmental samples including silage and fresh forage (Müller and Amend, 1997; Laser *et al.*, 2003; Kalač, 2012; Tangni *et al.*, 2013). Research undertaken *in vitro* on the correlation between heat produced, ergosterol content and biomass of *Penicillium roqueforti* has not yet been reproduced in environmental samples (Li *et al.*, 2009). However, a study by Auerbach (1996) examined the contribution that yeast had to the total ergosterol content in silage, during exposure to air. Extraction and analysis of ergosterol are time‐consuming and require the use of solvents for extraction, and are deficient in determining fungal biomass of specific genera. In addition, the plate count method that, because it relies on the proliferation of an organism via culture, does not represent the biomass within the sample (Lumsden *et al.*, 1990). Therefore, a method based on antibody detection that could detect differentiate between genera of fungi would be extremely beneficial.

Monoclonal antibodies (Mab) have been utilized extensively in the healthcare sector for therapeutic purposes (Waldmann, 1991) but have also been previously used to indicate the presence of fungi in the environment (Thornton, 2008a; Al‐Maqtoofi and Thornton, 2016; Sharpe *et al.*, 2016). This has been demonstrated by the tracking of *Trichoderma* species in compost‐based microcosms (Thornton, 2008b) and detection of *Fusarium* in hospital environmental samples (Al‐Maqtoofi and Thornton, 2016). Critically, Mab techniques allow for determination of biomass from the production of a standard calibration curve of the target organism and can be used to quantify changes in active growth of fungal species (Thornton, 2005). Monoclonal antibodies that can detect a range of fungi in the environment are available commercially and therefore present an opportunity in other sectors such as agriculture.

In the present study, we demonstrate the application of a previously described enzyme‐linked immunosorbent assay (ELISA) method (Thornton *et al.*, 1994) using both specific (JF5 for *Aspergillus* and *Penicillium*) and non‐specific (IE3 for pan‐ascomycete detection) Mabs to estimate fungal biomass and interrogate fungal growth dynamics during a laboratory incubation simulating the slow ingress of oxygen into silage. The aim was to assess the efficacy of Mab compared with ergosterol, as a positive control, as potential markers of fungal biomass in silage during aerobic deterioration under controlled conditions in aerobic exposure vessels. Furthermore, we speculate that this work could lead to the use of rapid diagnostic tools based on monoclonal antibody technology as on‐farm indicators of poor hygienic or nutritive quality of silage (Thornton, 2008a; Thornton *et al.*, 2012). To our knowledge, this is the first application of this technology to determine an estimate of active fungal biomass in silage.

## Results

### Effect of aerobic exposure on biochemical properties of silage

A controlled aerobic spoilage experiment was set up in 2.75 l aerobic exposure vessels (AEV) containing 750 g fresh weight silage from freshly opened bales (target density of 539 kg FM m^3^). AEVs were randomized within a controlled environment chamber, and each contained an individual temperature logger. At 0, 1, 2, 4, 8, 16 and 32 days, samples were destructively sampled and mixed well before analysis was undertaken.

One‐way analysis of variance (ANOVA) of 6 biological replicates indicated that there were biochemical changes in the silage temporally (from exposure to oxygen; Table 1). There was a difference for ethanol based on time point (*F*_6, 30_ = 4.43 *P* = 0.003). Ethanol production initially increased between 0 and 4 days (*M* = 7.06–9.06 g kg^−1^ DM, SD = 1.508–1.967) and had begun to decline by day 8 (*M* = 8.48 g kg^−1^ DM, SD = 0.866) until the final time point where the mean was 5.32 g kg^−1^ DM (SD 4.294). Similarly, propan‐1‐ol revealed a difference between time point (*F*_6,30_ = 2.46, *P* = 0.047). Propan‐1‐ol concentration increased from 0 day until 8 days where it was higher than the other days, where 16 days represented the lowest mean measurement (*M* = 0.04 g kg^−1^ DM, SD = 0.023). Heptanoic acid decreased from *M* = 0.07 g kg^−1^ DM on day 0, to 0.02 g kg^−1^ DM and remained stable until the final measurement of the experiment on day 32, and a difference based on time point (*F*_6,24_ = 6.97, *P* = < 0.001) was reported for this parameter. ANOVA reported no differences in the following measured parameters for time point: acetic acid, acetoin, propan‐1‐2‐diol, iso‐ and *n*‐butyric acid, propionic acid, iso‐ and *n*‐valeric, hexanoic acid, lactic acid, ash and pH. Mean results and SD for each time point for all chemical parameters are summarized in Table 1. DM varied between 28 and 33.1% in any given sample at any time point. Although there was a trend towards mean DM increasing towards the end of the experiment (0 day *M* = 28.76% SD = 3.819, day 32 M = 33.10%, SD = 4.454), there were no statistical differences observed, due to high variation seen between individual bales.

**Table 1:** Mean and Standard deviation values for key ensiling fermentation products at each time point through the 32 days aerobic exposure period of silage.

Analyte	0 days	1 day	2 days	4 days	8 days	16 days	32 days
pH							
Mean	4.9	4.8	4.8	4.7	4.7	4.7	5.0
SD	0.24	0.15	0.14	0.14	0.10	0.12	0.73
Ash (g kg^−1^ DM)							
Mean	89.0	87.2	91.2	87.7	92.2	89.9	91.3
SD	8.89	3.75	10.50	2.67	4.07	6.58	4.35
Dry matter %							
Mean	28.8	29.6	29.3	29.9	29.1	30.0	33.1
SD	3.82	3.80	3.45	3.09	2.83	4.78	4.45
Acetoin (g kg^−1^ DM)							
Mean	0.1	0.1	0.1	0.1	0.1	0.1	0.1
SD	0.03	0.04	0.05	0.02	0.05	0.05	0.03
Ethanol (g kg^−1^ DM)							
Mean	7.1	9.1	9.2	9.1	8.5	5.0	5.4
SD	1.51	1.59	2.95	1.97	0.87	2.11	4.29
Propan‐1‐ol (g kg^−1^ DM)							
Mean	0.1	0.1	0.1	0.1	0.1	0.0	0.1
SD	0.02	0.02	0.04	0.05	0.02	0.02	0.06
Propane‐1,2‐diol (g kg^−1^ DM)							
Mean	0.0	0.1	0.2	0.0	0.0	0.1	0.1
SD	0.00	0.18	0.28	0.06	0.01	0.08	0.19
Acetic acid (g kg^−1^ DM)							
Mean	8.5	6.7	5.5	6.1	7.0	8.7	7.7
SD	2.70	2.50	0.75	1.15	0.87	2.71	2.41
Isobutyric (g kg^−1^ DM)							
Mean	0.3	0.4	0.2	0.3	0.1	0.0	0.1
SD	0.27	0.37	0.13	0.45	0.04	0.03	0.09
*n*‐Butyric acid (g kg^−1^ DM)							
Mean	24.3	24.2	22.4	22.2	23.1	19.2	18.9
SD	5.07	5.35	8.21	6.99	3.90	2.75	4.93
Propionic acid (g kg^−1^ DM)							
Mean	1.7	1.6	1.3	1.3	1.1	0.8	0.9
SD	0.57	0.65	0.78	1.11	0.32	0.13	0.24
Isovaleric (g kg^−1^ DM)							
Mean	0.2	0.2	0.2	0.2	0.1	0.1	0.1
SD	0.07	0.06	0.07	0.11	0.04	0.02	0.01
*n*‐Valeric acid (g kg^−1^ DM)							
Mean	0.2	0.1	0.1	0.1	0.1	0.1	0.1
SD	0.07	0.07	0.08	0.16	0.05	0.03	0.03
Hexanoic acid (g kg^−1^ DM)							
Mean	0.5	0.4	0.3	0.4	0.3	0.3	0.2
SD	0.27	0.27	0.25	0.56	0.16	0.08	0.07
Heptanoic acid (g kg^−1^ DM)							
Mean	0.1	0.1	0.0	0.0	0.0	0.0	0.0
SD	0.02	0.05	0.02	0.02	0.01	0.02	0.01
Lactic acid (g kg^−1^ DM)							
Mean	38.1	34.6	32.0	34.7	36.2	39.7	36.3
SD	5.16	7.43	2.81	6.48	3.98	7.52	12.19
Ammoniacal nitrogen (g kg^−1^ DM)							
Mean	4.2	4.1	4.4	3.8	3.9	4.3	4.4
SD	0.72	0.70	0.96	0.63	0.46	0.63	0.92

### Comparison of indicators of fungal biomass

**Fig. 1:**
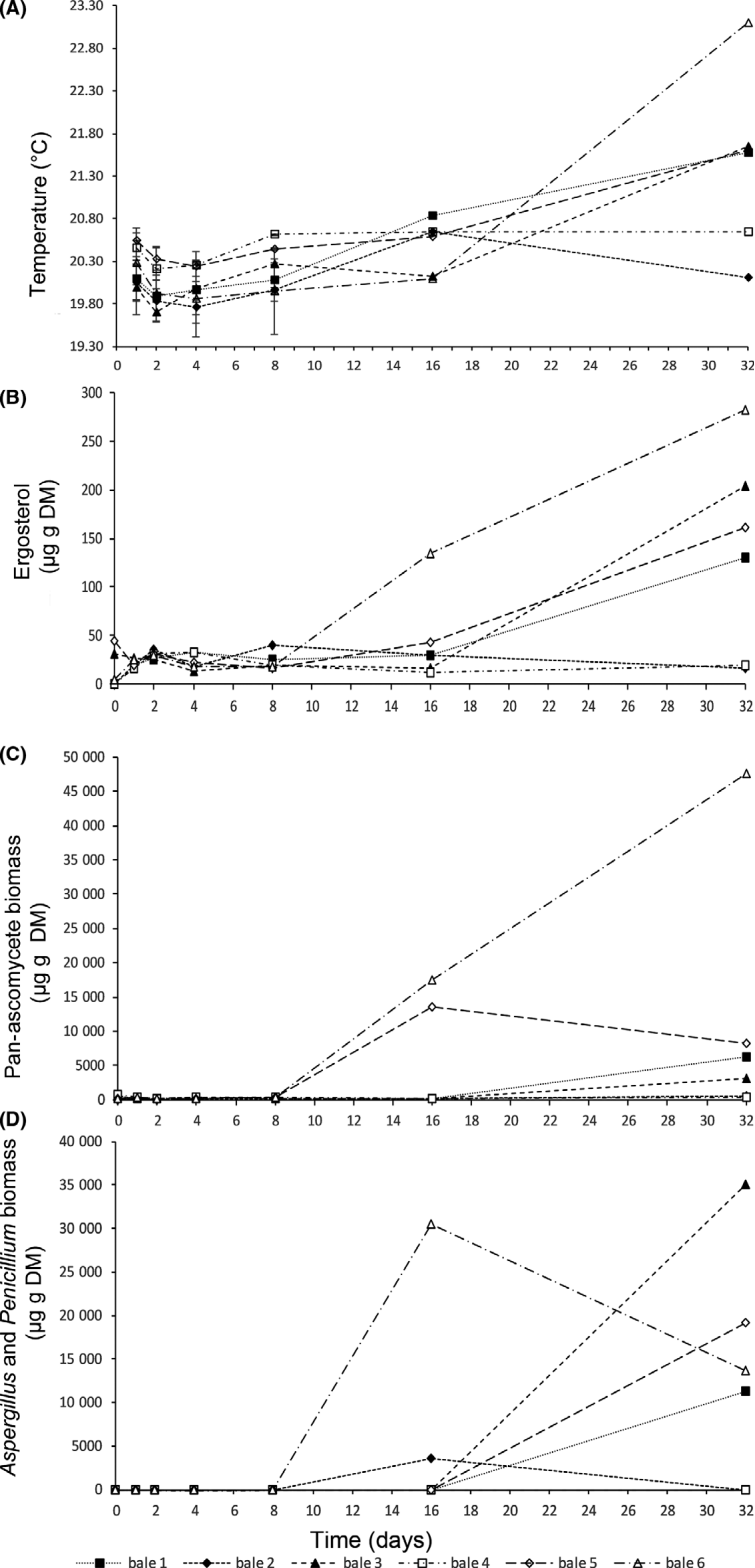
Aerobic deterioration of silage over 32 days as measured by (A) temperature, (B) ergosterol content, (C) determination of fungal growth using IE3, a pan‐ascomycete antibody, (D) determination of fungal growth using JF5, an antibody specific for *Aspergillus* and *Penicillium *spp. Each point at a given timepoint represents a biological replicate originating from one of six bales.

Over the 32 days period of aerobic spoilage bale 6 increased in temperature to 23.1°C (Fig. 1, Table 2). Bales 1, 3 and 5 reached 21.58°C, 21.64°C and 21.6°C respectively. Bales 2 and 4 remained at ambient temperature, indicating that aerobic spoilage did not occur within these bales. Temperature increase was only observed on day 32 of measurements, for specific bales. One‐way ANOVA and Tukey post hoc analysis revealed that day 32 was the only time point statistically different from the rest of the time points (day 0–16; *F*_6,41_ = 8.50, *P* = < 0.01). Ergosterol measurements increased with exposure to aerobic conditions in 4 of the 6 bales. At time point 0, three of the six bales had levels that were undetectable by HPLC, indicating a low fungal load in the starting material. Ergosterol increased in bale six after day 8 and continued to increase reaching 282.8 µg g^−1^ DM on day 32. An increase was observed at day 32 in bale 1, 3 and 5 with no increase observed in bales 2 and 4. The average ergosterol increased from day 8, due to one replicate (bale 6), increasing in ergosterol from this point forwards. At time 16 and 32, this bale continued to report the highest ergosterol load for the group. Bales 1, 3 and 5 increased in ergosterol load from d 16 with bales 2 and 4 although increasing between 0 and 4 remained below 40 µg g^−1^ DM through the remainder of the experimental period. One‐way ANOVA for the bales as a group reported a statistical difference between time points (*F*_6,41_ = 6.513, *P* = < 0.01), and Tukey post hoc analysis indicated grouping of measurements at day 0 and 16–32 (Fig. 1B). Fungal biomass as measured quantitatively by IE3 (pan‐ascomycete) antibody and JF5 (*Aspergillus* and *Penicillium* specific) was tested statistically with time point as the main factor using ANOVA. This analysis revealed a statistical difference (*F*_6,41_ = 4.54, *P* = 0.002) for pan‐ascomycete biomass and (*F*_6,41_ = 7.63, *P* = < 0.001) for *Aspergillus* and *Penicillium* biomass with Tukey post hoc 95% confidence intervals test revealing the same two groups in the mean results with day 0, 1, 2, 4 and 8 being statistically different from means at day 32, with day 16 not statistically differentiated from either group (Table 2). The individual measurements for each bale are presented in Figure 1C. There were some distinct differences within the data set with bales 5 and 6 showing an increase in fungal biomass from day 8. Biomass in bale 6 continued to increase until day 32, whereas fungal biomass in bale 5 dropped at the final time point. Bale 1 and 3 showed an increase from 160 and 90 to 6240 and 3030 µg g^−1^ DM in biomass at day 16–32. No increase in biomass was observed in bales 2 and 4 for any of the methods where the maximum recorded biomass was 740 µg g^−1^ DM (Table 2). Although results obtained with antibody JF5 (Fig. 1D) showed a pattern of increase in *Aspergillus* and *Penicillium* in line with the results gained with IE3, the estimated biomass shows a discrepancy of approximately 15 mg g^−1^. Pearson’s test of correlation between the methods of detection was used to determine whether there was a linear association between the four methods (Table 3). Notably, there was a positive association between temperature and ergosterol (*r* (41) = 0.84, *P* = < 0.001) and temperature and pan‐fungal biomass (*r* (41) = 0.73, *P* = < 0.001) and a weaker positive linear relationship between temperature and *Aspergillus* and *Penicillium* biomass (*r* (41) = 0.56, *P* = < 0.001), and this corroborates the pattern of aerobic spoilage identified in Figure 1. There was also a positive association between ergosterol and both measures of fungal biomass, with correlation coefficients of (*r* (41) = 0.82, *P* = < 0.001) and (*r* (41) = 0.80, *P* = < 0.001) for *Aspergillus* and *Penicillium* biomass and pan‐ascomycete biomass respectively.

**Table 2:** Numerical values for temperature, ergosterol concentration and fungal biomass as determined by IE3 (pan‐ascomycete antibody) or JF5 (*Aspergillus* and *Penicillium* specific antibody) over a 32 days aerobic deterioration period. Mean values represent the average of six biological replicates which were destructively sampled.

	Time (days) Bale	0	1	2	4	8	16	32
Temperature (°C)	1	n/a	20.10	19.90	19.96	20.08	20.83	21.58
	2	n/a	20.05	19.83	19.75	19.96	20.64	20.11
	3	n/a	20.00	19.71	19.98	20.26	20.12	21.64
	4	n/a	20.45	20.22	20.25	20.62	20.65	20.64
	5	n/a	20.55	20.33	20.24	20.44	20.58	21.60
	6	n/a	20.28	19.92	19.86	19.95	20.10	23.10
	Average	n/a	20.24^a^	19.98^a^	20.01^a^	20.22^a^	20.49^a^	21.45^b^
	SD	n/a	0.23	0.24	0.20	0.27	0.30	1.03
Ergosterol (µg g^−1^ DM)	1	0	18.9	26.7	33.3	25.2	29.4	130.1
	2	0	19.3	34.9	17.5	39.4	29.4	16.5
	3	31.2	26.4	24.5	13.7	19.3	15.9	204.1
	4	0	16.7	31.7	32.5	19.7	12	19.9
	5	44	19.9	31.1	22.1	16.2	43.3	161.9
	6	4	24.8	29	17.2	17.8	134.7	282.8
	Average	26.4^a^	21^a^	29.7^ab^	22.7^a^	22.9^a^	44.1^ab^	135.9^b^
	SD	20.40	3.80	3.70	7.30	8.60	45.70	104.60
Pan‐ascomycete biomass (IE3; µg g^−1^ DM)	1	460.0	150.0	70.0	120.0	80.0	160.0	6240.0
	2	30.0	110.0	130.0	80.0	70.0	80.0	520.0
	3	250.0	70.0	90.0	390.0	90.0	90.0	3030.0
	4	740.0	260.0	120.0	350.0	250.0	90.0	290.0
	5	90.0	180.0	40.0	130.0	330.0	13620.0	8220.0
	6	220.0	270.0	200.0	180.0	320.0	17480.0	47520.0
	Average	300.0^a^	170.0^a^	110.0^a^	210.0^a^	190.0^a^	5250.0^ab^	10970.0^b^
	SD	260.0	80.0	50.0	130.0	120.0	8070.0	18180.0
*Aspergillus* and *Penicillium* biomass (JF5; µg g^−1^ DM)	1	0.05	0.05	0.06	7.72	0.03	0.12	11332.64
	2	1.61	0.05	0.06	0.06	0.06	3609.29	1.93
	3	0.01	0.48	0.24	0.96	0.03	0.06	35021.22
	4	6.43	1.93	0.06	0.02	0.06	0.48	3.86
	5	0.03	0.03	0.02	0.06	0.06	0.48	19106.45
	6	0.05	7.72	0.03	0.06	0.03	30429.95	13592.56
	Average	1.40^a^	1.70^a^	0.10^a^	1.50^a^	0.00^a^	5673.40^ab^	13176.40^a^
	SD	2.60	3.00	0.10	3.10	0.00	12213.80	13137.90

Groups which do not share a letter are not significant (*P* > 0.05).

**Table 3:** Summary of Pearson correlation coefficient (*r* values) comparing data at all time points from four parameters (against each other temperature, ergosterol concentration, fungal biomass as determined by IE3 (a pan‐ascomycete) or JF5 (*Aspergillus* & *Penicillium* specific) antibody).

	*Aspergillus* & *Penicillium* biomass	Ergosterol	Pan‐fungal biomass
Temperature			
*r*	0.5612	0.8394	0.7267
*P* value	< 0.001	< 0.001	< 0.001
Pan‐fungal biomass			
*r*	0.479	0.8027	
*P* value	0.0013	< 0.001	
Ergosterol			
*r*	0.8188		
*P* value	< 0.001		

Additional measurements were taken by specific Mab that selectively bind to antigens expressed by fungi in the genus *Fusarium* (ED7), *Trichoderma* (MF2) or *Candida* (MC3). Quantitative data showed no detectible presence of *Fusarium*, *Trichoderma or Candida*, with all readings under the threshold of minimum detectible level using this method (data not shown).

## Discussion

We investigated the suitability of an ELISA method previously described for use in soil and environmental samples for detecting the progression of aerobic spoilage, with a focus on the proliferation of filamentous fungal biomass and the population dynamics of key organisms involved in aerobic spoilage within the total population.

### Individual bales from the same batch varied in their response to aerobic exposure

It was found that limited exposure to aerobic conditions had a variable effect on silage that had been sampled from the same batch of individual bales. Silage quality and aerobic stability can be affected by factors such as the number of layers of wrap, quality of the seal and damage to plastic wrap when in storage, so this was not unexpected (Keller *et al.*, 1998). The packing density meant that the ingress of oxygen to the packed silage was slow compared with a traditional aerobic stability experiment, and this mimics the ingress at the face of a clamp or vertically lower regions of the clamp where CO_2_ concentrations are still elevated and thus reduce the ability of fungi to grow. This was reflected in the chemical profiles of the silage over 32 days. The increase in ethanol seen between day 0 and 2 is representative of the growth of yeasts or heterofermentative lactic acid bacteria that ferment sugars to ethanol and CO_2_ (Pahlow *et al.*, 2003).

### Chemical parameters of silage reveal stable profile during time of aerobic exposure

DM percentage of between 28 % and 29 % is considered high enough for an adequate silage preservation and is around the national long‐term average for grass silage produced in the UK (Genever, 2011). The ash content was relatively high at 89 g kg^−1^ DM with recommendations of less than 80 g kg^−1^ DM for good ensilage but with an upper limit of 100 g kg^−1^ DM for adequate preservation, which may reflect a degree of soil contamination (McDonald *et al.*, 1991). The pH values are higher than expected at the % DM content of the silage, due to the fact that it was made in bales (which are known to have a restricted fermentation compared with clamp silage), and there was evidence of secondary fermentation (Table 1). At opening, the fermentation acids indicate a poor preservation in all bales with relatively low levels of the beneficial preservation lactic acid and high levels of acetic and butyric acid. The ratio of lactic to the sum of the other acids was approaching 1:1. Acetic and butyric acid are known inhibitors of fungal growth which may have contributed to the inhibited fungal proliferation in some samples (Kung Jr *et al.*, 2018). Concentrations of ethanol were also slightly elevated compared to a well‐preserved silage, taken together the evidence suggests that one or more of the following groups: enterobacteria, *Clostridia*, heterofermetative lactic acid bacteria or yeast populations were active during the preservation process (Kung Jr *et al.*, 2018).

Aerobic exposure vessels were packed to mimic the clamp face (530 kg FM m^3^) where there is a slow ingress of oxygen compared with a traditional aerobic stability experiment where AEVs are loosely packed (Williams and Shinners, 2012; Da Silva *et al.*, 2015). Therefore, there are smaller than changes compared to traditional aerobic stability experiments in the fermentation acids and alcohols through the 32 day time‐course of aerobic challenge (Davies *et al.*, 1996). This is likely to have been due to the limited ingress of oxygen as a result of the packing density, which is representative of the clamp face. For % DM content in the last sample time point, there is an increase, which can be explained by water loss from the surface of the silage during the extended period of aerobic exposure. It also coincides with increase in temperature in 2 replicates of silages (see Fig. 1) which is an indicator of aerobic deterioration. Except for pH and *n*‐butyric acid, there is little evidence to suggest that the changes in chemical composition are anything other than natural variation in replicates. The change in ethanol is likely due to the activity of organisms such as heterofermentative lactic acid bacteria or microorganisms growing in facultative anaerobic growth mode such as enterobacteria and yeasts due to the packing density (Pahlow *et al.*, 2003). In addition, the higher levels of butyric acid found in the silage will reduce the speed of the microorganisms associated with aerobic deterioration.

### Suitability of monoclonal antibodies for predicting fungal load

Fungal growth (population) dynamics within silage undergoing aerobic spoilage were determined by ergosterol levels and compared temperature with the novel application of a previously described ELISA Mab method. Ergosterol and Mabs showed the same pattern of fungal proliferation across the six replicates, which was detectable at the same time points. Pan‐ascomycete biomass had a better correlation with ergosterol and temperature than the *Aspergillus* and *Penicillium* specific Mab as it continued to increase at the final time point at 32 days. A possible explanation for JF5 Mab not responding in exactly the same way could be the proliferation of non‐target fungi that are commonly detected in silage and detected by IE3 but not JF5, such as *Cladosporium, Monascus*, *Mucor, Acremonium*, *Mycelia sterilia*, *Rhizopus*, *Paecilomyces* and *Scopulariopsis* spp. (Alonso *et al* *.*, 2013). Alternatively, as JF5 does not detect the development of conidia, it could indicate sporulation by the fungus and a drop in active biomass. Increase in temperature as an indication of aerobic spoilage lagged behind both ergosterol and Mab leading to the conclusion that it is a less reliable determinant of aerobic spoilage because more spoilage could have occurred before detection. In addition, heat can dissipate from a sample before detection resulting in underrepresentation of aerobic spoilage. Therefore, Mab technology has potential to be developed as an on‐farm diagnostic method (Thornton, 2008). The possible presence of exocellular ergosterol and the slow breakdown of ergosterol in dead fungal biomass mean that ergosterol concentration may not be accurate in determining currently active fungal biomass in environmental samples (Zhao *et al.*, 2005). It has not been determined if this is the case in the Mab method as the speed of breakdown of the antigens after death of fungal cells has not yet been documented. However, within silage this characteristic could still be a useful determinant of historic spoilage, which would still indicate a loss of nutritive quality.

Issues with ergosterol abundance as a fungal biomass marker are not novel and have been previously discussed within the literature, the main criticism being that ergosterol may be differentially abundant in different fungal species or at different growth stages (Tothill *et al.*, 1992), and it degrades under UV exposure (Mille‐Lindblom *et al.*, 2004). Antigen detection may present the same issues. They could be mitigated to a certain degree if calibration of known biomass is undertaken with the fungal strain identified within the sample under similar conditions. Use of specific antibodies can offer information on the presence or absence of a particular fungal genus, as shown by the lack of detection from the *Fusarium* and *Trichoderma* specific antibodies in the present study, indicating that they did not proliferate under these conditions in these sample types. This could be of value to studies investigating mycotoxin production by fungi in silage. Although the method was not successful in positively identifying the presence of *Candida *spp. through detection with a specific antibody (MC3 see Table S1), this does not negate the presence of other yeasts, which are known to have an important role in the aerobic deterioration of silage. As such, this is an area that warrants further research (Rossi and Dellaglio, 2007; O'Brien *et al.*, 2008; Carvalho *et al.*, 2016). In addition, Mabs may also differ in their ability to detect active fungal biomass. It is possible that the discrepancy observed between biomass detected by IE3 and JF5 represents a difference in sensitivity between the antibodies, as reported by Al‐Maqtoofi and Thornton (2016) in *Fusarium* Spp. Using a specific antibody and an increase in knowledge of fungal isolates from silage could help to refine the calibration of this method. The Mab JF5 detects growing mycelium but not un‐germinated conidia (Thornton, 2008a); therefore, it represents the hyphal biomass of *Aspergillu*s and *Penicillium* at time of sampling, which is a key advantage of this method over others described; however, this trait may differ between antibodies depending on expression of the target antigen and has yet to be reported for the range of antibodies used in this study.

This Mab approach also gave us an insight into the growth dynamics of fungi that have a key role in the aerobic spoilage of silage. This contributes to our understanding into the monitoring and detection of fungi across a silage clamp or within bales. There is no current threshold for determining if silage is safe to feed to livestock. The experiment, though limited in the range of fungi observed, indicated the clear variance between individual bales and that silage quality does vary considerably within a single batch, and therefore, a rapid method to determine fungal biomass could be of considerable value to the agricultural industry in developing threshold values for silage quality.

## Experimental procedures

### Experimental design and sampling

In May 2017, six bales of grass (predominately *Lolium perenne* permanent pasture) silage were randomly selected from the same batch, originating from the Rothamsted Research Farm (North Wyke, Devon, UK) after 9 months of ensilage. The purpose of the experiment was to test the efficacy of the monoclonal antibody‐based ELISA to detect changes in aerobic stability within silage. Because high variation is known to occur between individual bales of forage ((O'Brien *et al.*, 2006), the number of bales (biological replicates) was maximized, to ensure greatest chance of capturing variability. Fields were mown on 4 August 2016 to leave a stubble height of 7 cm and wilted for 24 h before baling. Bales were wrapped in 4 layers of plastic (750 Green 25 µm, ®Silotite, RPC bpi agriculture, Leominster, UK). Bales were stored in a stack, horizontally 3 high on a concrete base until selection.

Prior to sampling, bales were removed from the storage stack and placed separately on their end under cover. Sampling was undertaken using a mechanical silage corer (Dairy One forage Lab, Ithaca, NY, USA), following the sampling strategy as described in (O'Brien *et al.*, 2006), to ensure a homogenous sample from each bale. Briefly, cores were taken from predetermined, spatially discrete sampling points on the bale from the full circumference of the round edge resulting in a total of 7 kg FM of silage from each bale. Equipment was cleaned with ethanol, and gloves were changed between handling samples from each bale. Cores were mixed thoroughly to produce a homogenous representative sample of the bale and split into six identical replicates that were placed in individual aerobic exposure vessels (plastic tubes with tops open to the atmosphere measuring 15 cm high, 15 cm diameter; AEV) and destructively sampled at six time points during the experiment. AEV was cleaned with ethanol prior to packing with 750 g FM, a density of 530 kg FM m^3^. For each bale, 1 AEV was designated for each destructive sampling time point (0, 1, 2, 4, 8, 16 and 32 days after the start of the experiment). A temperature data logger was placed in the centre of each sample. AEVs were randomized within a controlled temperature environment chamber which was set at 20°C ± 0.5 and ambient humidity. At each destructive sampling, samples were thoroughly mixed and approximately 450 g FM was subsampled for determination of dry matter (DM) and ash (100 g), pH (10 g), ELISA (10 g), volatile fatty acids (VFAs), lactic acid and ammonia (50 g), ergosterol analysis (as described below) near infrared spectroscopy (NIRS) and an archive sample (300 g FM) which was vacuum packed and frozen at −20°C.

### Nutritional quality assessment

Approximately 50 g FM was placed in an oven at 80°C for *ca.* 24 h until no further loss of weight was recorded. These samples were then milled through a 1 mm screen and 3 g DM furnaced, at 505 °C for 12 h (ramp rate 2°C min^−1^) leaving only the ash from which the organic matter (OM) was calculated by mass difference. The pH was determined by measuring a supernatant prepared by agitating 10 g FM with 90 ml milli‐Q water and incubated at room temperature for 10 min using a pH electrode (Jenway 3320, Cole Palmer, Staffordshire, UK). Analysis of VFAs was carried out on a water extract prepared using 10 g FM and 90 ml of distilled water that was stored at 4°C for 16 h before being filtered, and the filtrate used for subsequent analysis. This was used to determine the VFAs and alcohols by the gas chromatography method as described by (Zhu *et al.*, 1996). Lactic acid was determined by a HPLC method as described by (Canale *et al.*, 1984).

### Enzyme‐linked immunosorbent assay

To immobilize the antigens, 0.5 g DM of finely ground (1 mm) silage from each sample was placed in a universal tube with 5 ml phosphate‐buffered saline (PBS) and agitated for 1 h. A 1.5 ml aliquot was centrifuged at 15 700 × *g* for 10 min. The supernatant (100 µl) was added to well 1 of each sample’s dilution series on a 96‐well microtitre plate (Nunc immuno‐plate maxisorb^®^, Thermo Fisher 168 Third Avenue Waltham, MA USA 02451). Subsequent double dilutions were performed with 50 µl of PBS for a total of 12 wells, resulting in each sample occupying 24 wells in total (2 rows) with the first well containing solely 50 µl supernatant and the last solely 50 µl PBS. A positive control, consisting of known biomass of freeze‐dried mycelium corresponding to the target genus, and a negative (PBS) control were also included. Mycelium for positive controls was cultured from laboratory stocks (*Aspergillus fumigatus*, *Candida albicans, Fusarium solani, Trichoderma hamatum)* by culturing single species in 100 ml potato dextrose broth for 5 days, strained through sterile Miracloth (Calbiochem^®^, VWR Hunter Boulevard, Magna Park, Lutterworth, Leicestershire, England) and rinsing in sterile water and snap‐freezing in liquid nitrogen before freeze‐drying. Plates were incubated in sealed plastic bags at 4°C for 16 h. Unbound material was removed by washing 3 times with PBS containing 0.05% (v/v) Tween‐20 [polyoxyethylene sorbitan monolaurate (PBST)], then once with PBS and finally once with dH_2_O. All washes were undertaken at 5 min intervals. Plates were then dried in a laminar flow hood under sterile conditions and stored at 4°C in sealed plastic bags until the ELISA was performed. To quantify bound antigen, 50 µl of the Mab IE3 (pan‐ascomycete), JF5 (*Aspergillus* and *Penicillium*), MC3 (*Candida *spp.) or ED7 (*Fusarium *spp.; Isca Diagnostics, Exeter, UK; Table S1) was added to each well and incubated at 4°C for 1 h in sealed plastic bags. Plates were washed with 4 PBST washes at 5 min intervals before adding 50 µl of 1:1000 dilution of the peroxidase‐conjugated goat anti‐mouse polyvalent secondary antibody (Sigma‐Aldrich Company, Dorset, England) to each well and incubated in ambient conditions in sealed plastic bags for 1 h. This was followed by 3 PBST washes and 1 PBS wash at 5 min intervals. Bound antibody levels were then visualized using a tetramethylbenzidine substrate solution (5 ml milli‐Q water, 5 ml 0.2 M C_2_H_5_ONa, 195 µl 0.2 M Na_3_C_6_H_5_O_7_, 5 µl 30 % H_2_O_2_, 100 µl TMB 10 mg ml^−1^ in DMSO) incubated for 30 min at room temperature. This reaction was stopped by adding 50 µl 3 M H_2_SO_4_ to each well. The absorbance values were then determined at 450 nm with an automatic plate reader. A calibration curve was plotted from the wells containing a known fungal biomass, and the absorbance values were used to calculate the fungal biomass present in silage samples.

### Ergosterol measurements

Ergosterol was extracted from silage according to the method described by (Rousk and Bååth, 2007). Ground material (0.5 g DM) was mixed with 2 ml cyclohexane and 8 ml 10 % KOH which was vortexed for 10 s. Sonication was carried out in a heated water bath at 25°C for 25 min before incubating at 70°C for 90 min in a heat block and addition of 2 ml MQ. An additional 2 ml cyclohexane was added before a 5 min centrifugation (2284 × *g*). The upper phase of cyclohexane was extracted into a test tube, and then, extraction was repeated a further two times with 4 ml cyclohexane to the remaining sample, vortexed for 10 s and centrifuged at 2285 × *g* for 5 min; then, upper phase was extracted into the same test tube as the first. The extracted cyclohexane was evaporated until dry under N_2_ at 40°C and stored at 4°C in the dark. Samples were re‐suspended by adding 750 µl MeOH with glass syringe and vortexed to dissolve. Extracts were filtered through 0.45 µm sterile syringe filters (VWR international Ltd, Lutterworth, Leicestershire) syringe and run on HPLC Agilent Zorbax Rx‐C18 Column part number 866967‐902 at 1 min ml^−1^ flow rate (Mobile phase H_2_O:MeOH: 2:98 UV Absorption 282 nm).

### Statistical analysis

Statistical analysis was conducted using Genstat 18th edition (VSN International, 2015). One‐way analysis of variance (ANOVA) was used to compare analytes across time points with Tukey post hoc tests indicating which groups were statistically different. Where residuals were skewed, log‐transformation was carried out, and residual distributions were checked for normality. To determine the strength of a linear relationship between variables, Pearson’s correlation coefficients were calculated. Differences were considered significant when *P* values were < 0.05.

## Conflict of interest

None declared.

## Author contributions

KLC, CJH, DD and ML secured the funding. KLC, CJH and DD designed the experiment. BB, CJH, JM and KLC carried out the experimental work, supported by DD. BB, CH and KLC carried out the ergosterol extractions. KLC and DD wrote the manuscript. All authors contributed to revisions.

## Supporting information


**Table S1**. Summary information for each antibody used in the study.

## References

[mbt213552-bib-0001] Al‐Maqtoofi, M. , and Thornton, C.R. (2016) Detection of human pathogenic *Fusarium* species in hospital and communal sink biofilms by using a highly specific monoclonal antibody. Environ Microbiol 18: 3620–3634. 26914362 10.1111/1462-2920.13233

[mbt213552-bib-0002] Alonso, V.A. , Pereyra, C.M. , Keller, L.A. , Dalcero, A.M. , Rosa, C.A. , Chiacchiera, S.M. , and Cavaglieri, L.R. (2013) Fungi and mycotoxins in silage: an overview. J Appl Microbiol 115: 637–643. 23445404 10.1111/jam.12178

[mbt213552-bib-0003] Auerbach, H. (1996) Verfahrensgrundlagen zur Senkung des Risikos eines Befalls von Silagen mit *Penicillium roqueforti* und einer Kontamination mit Mykotoxinen dieses Schimmelpilzes: FAL. Landbauforschung Völkenrode, Issue: Sonderheft 168: 1–167.

[mbt213552-bib-0004] Auerbach, H. , Oldenburg, E. , and Weissbach, F.J. (1998) Incidence of *Penicillium roqueforti* and roquefortine C in silages. J Sci Food Agric 76: 565–572.

[mbt213552-bib-0005] Bolsen, K. , Ashbell, G. , and Weinberg, Z. (1996) Silage fermentation and silage additives‐review. Asian‐Australas J Anim Sci 9: 483–494.

[mbt213552-bib-0006] Borreani, G. , and Tabacco, E. (2010) The relationship of silage temperature with the microbiological status of the face of corn silage bunkers. J Dairy Sci 93: 2620–2629. 20494171 10.3168/jds.2009-2919

[mbt213552-bib-0007] Bueno, D.J. , Silva, J.O. , and Oliver, G. (2004) Fungal isolation and enumeration in foods In Public Health Microbiology. Spencer J.F.T. and Ragout de Spencer A.L. (eds.). Totowa, NJ: Humana Press Inc., pp.127–131. 10.1385/1-59259-766-1:127 15156024

[mbt213552-bib-0008] Cafarchia, C. , Figueredo, L.A. , and Otranto, D. (2013) Fungal diseases of horses. Vet Microbiol 167: 215–234. 23428378 10.1016/j.vetmic.2013.01.015

[mbt213552-bib-0009] Canale, A. , Valente, M.E. , and Ciotti, A. (1984) Determination of volatile carboxylic acids (C1–C5i) and lactic acid in aqueous acid extracts of silage by high performance liquid chromatography. J Sci Food Agric 35: 1178–1182.

[mbt213552-bib-0010] Carvalho, B. , Ávila, C. , Krempser, P. , Batista, L. , Pereira, M. , and Schwan, R. (2016) Occurrence of mycotoxins and yeasts and moulds identification in corn silages in tropical climate. J Appl Microbiol 120: 1181–1192. 26787003 10.1111/jam.13057

[mbt213552-bib-0011] Cheli, F. , Campagnoli, A. , and Dell'Orto, V. (2013) Fungal populations and mycotoxins in silages: from occurrence to analysis. Anim Feed Sci Technol 183: 1–16.

[mbt213552-bib-0012] Coblentz, W. , and Akins, M. (2018) Silage review: Recent advances and future technologies for baled silages. J Dairy Sci 101: 4075–4092. 29685278 10.3168/jds.2017-13708

[mbt213552-bib-0013] Da Silva, T. , Smith, M. , Barnard, A. , and Kung, L. Jr (2015) The effect of a chemical additive on the fermentation and aerobic stability of high‐moisture corn. J Dairy Sci 98: 8904–8912. 26454298 10.3168/jds.2015-9640

[mbt213552-bib-0014] Davies, D.R. , Merry, R.J. , Jones, R. , and Fychan, R. (1996) Effect of different additives on the aerobic stability of ensiled whole crop maize In Proc. 11th Int. Silage Conf., Univ. Wales. Aberystwyth, UK. Jones D.I.H., Jones R., Dewhurst R., Merry R.J., and Haigh P.M. (eds.). Aberystwyth, UK: Cambrian Press, pp. 264–265.

[mbt213552-bib-0015] Djajakirana, G. , Joergensen, R. , and Meyer, B. (1996) Ergosterol and microbial biomass relationship in soil. Biol Fertil Soils 22: 299–304.

[mbt213552-bib-0016] Fink‐Gremmels, J. (2008) Mycotoxins in cattle feeds and carry‐over to dairy milk: a review. Food Additives Contamin 25: 172–180. 10.1080/02652030701823142 18286407

[mbt213552-bib-0017] Genever, L. (2011) Making grass silage for better returns. In AHDB report. Huntingdon, UK: AHDB.

[mbt213552-bib-0018] Gessner, M.O. (2005) Ergosterol as a measure of fungal biomass In Methods to Study Litter Decomposition. Berlin, Germany: Springer, pp. 189–195.

[mbt213552-bib-0019] Gessner, M.O. , Bauchrowitz, M.A. , and Escautier, M. (1991) Extraction and quantification of ergosterol as a measure of fungal biomass in leaf litter. Microb Ecol 22: 285–291. 24194343 10.1007/BF02540230

[mbt213552-bib-0020] Jonsson, A. (1991) Growth of *Clostridium tyrobutyricum* during fermentation and aerobic deterioration of grass silage. J Sci Food Agric 54: 557–568.

[mbt213552-bib-0021] Kalač, P. (2012) Carotenoids, ergosterol and tocopherols in fresh and preserved herbage and their transfer to bovine milk fat and adipose tissues: a review. J Agrobiol 29: 1–13.

[mbt213552-bib-0022] Keller, T. , Nonn, H. , and Jeroch, H. (1998) The effect of sealing and of additives on the fermentation characteristics and mould and yeast counts in stretch film wrapped big‐bale lucerne silage. Arch Anim Nutr 51: 63–75. 10.1080/17450399809381906 9638306

[mbt213552-bib-0023] Keller, L. , Pereyra, M.G. , Keller, K. , Alonso, V. , Oliveira, A. , Almeida, T. , *et al* (2013) Fungal and mycotoxins contamination in corn silage: Monitoring risk before and after fermentation. J Stored Prod Res 52: 42–47.

[mbt213552-bib-0024] Kung, L. (2011) Silage temperatures: how hot is too hot? URL https://cdn.canr.udel.edu/wp-content/uploads/2014/02/HowHotisTooHot-2011.pdf.

[mbt213552-bib-0025] Kung Jr, L. , Shaver, R. , Grant, R. , and Schmidt, R. (2018) Silage review: Interpretation of chemical, microbial, and organoleptic components of silages. J Dairy Sci 101: 4020–4033. 29685275 10.3168/jds.2017-13909

[mbt213552-bib-0026] Laser, H. , Boberfeld, W.O.V. , Wöhler, K. , and Wolf, D. (2003) Effects of the botanical composition and weather conditions on mycotoxins in winter forage from grassland. Mycotoxin Res 19: 87–90. 23604677 10.1007/BF02940101

[mbt213552-bib-0027] Li, Y. , Wadsö, L. , and Larsson, L. (2009) Impact of temperature on growth and metabolic efficiency of *Penicillium roqueforti*–correlations between produced heat, ergosterol content and biomass. J Appl Microbiol 106: 1494–1501. 19210571 10.1111/j.1365-2672.2008.04110.x

[mbt213552-bib-0028] Lindgren, S. , Pettersson, K. , Jonsson, A. , Lingvall, P. , and Kaspersson, A. (1985) silage inoculation ‐ selected strains, temperature, wilting and practical application. Swed J Agric Res 15: 9–18.

[mbt213552-bib-0029] Lumsden, R. , Carter, J. , Whipps, J. , and Lynch, J. (1990) Comparison of biomass and viable propagule measurements in the antagonism of *Trichoderma harzianum* against *Pythium ultimum* . Soil Biol Biochem 22: 187–194.

[mbt213552-bib-0030] March, M. , Haskell, M. , Chagunda, M. , Langford, F. , and Roberts, D. (2014) Current trends in British dairy management regimens. J Dairy Sci 97: 7985–7994. 25306285 10.3168/jds.2014-8265

[mbt213552-bib-0031] McDonald, P. , Henderson, A. , and Heron, S. (1991) The biochemistry of silage, 2nd ed Marlow J. (ed.). Kent, UK: Chalcombe Publications.

[mbt213552-bib-0032] Mille‐Lindblom, C. , von Wachenfeldt, E. , and Tranvik, L.J. (2004) Ergosterol as a measure of living fungal biomass: persistence in environmental samples after fungal death. J Microbiol Methods 59: 253–262. 15369861 10.1016/j.mimet.2004.07.010

[mbt213552-bib-0033] Muck, R.E. (2013) Recent advances in silage microbiology. Agr Food Sci 22: 3–12.

[mbt213552-bib-0034] Muck, R. , and Pitt, R.E. (1994) Aerobic deterioration in corn silage relative to the silo face. Trans Am Soc Agric Eng 37: 735–743.

[mbt213552-bib-0035] Muck, R. , Pitt, R. , and Leibensperger, R.J. (1991) A model of aerobic fungal growth in silage. 1. Microbial characteristics. Grass Forage Sci 46: 283–299.

[mbt213552-bib-0036] Müller, H.M. , and Amend, R. (1997) Formation and disappearance of mycophenolic acid, patulin, penicillic acid and PR toxin in maize silage inoculated with Penicillium roqueforti. Arch Anim Nutr 50: 213–225. 10.1080/17450399709386133 9272220

[mbt213552-bib-0037] Nicolaisen, M. , Supronienė, S. , Nielsen, L.K. , Lazzaro, I. , Spliid, N.H. , and Justesen, A.F. (2009) Real‐time PCR for quantification of eleven individual Fusarium species in cereals. J Microbiol Methods 76: 234–240. 19047000 10.1016/j.mimet.2008.10.016

[mbt213552-bib-0038] Niessen, L. (2007) PCR‐based diagnosis and quantification of mycotoxin producing fungi. Int J Food Microbiol 119: 38–46. 17804102 10.1016/j.ijfoodmicro.2007.07.023

[mbt213552-bib-0039] O'Brien, M. , O Kiely, P. , Forristal, P. , and Fuller, H. (2006) A note on sampling baled grass silage for fungal propagules. J Anim Feed Sci 15: 305.

[mbt213552-bib-0040] O'Brien, M. , O’Kiely, P. , Forristal, P.D. , and Fuller, H.T. (2007) Quantification and identification of fungal propagules in well‐managed baled grass silage and in normal on‐farm produced bales. Anim Feed Sci Tech 132: 283–297.

[mbt213552-bib-0041] O'Brien, M. , O’Kiely, P. , Forristal, P. , and Fuller, H. (2008) Fungal contamination of big‐bale grass silage on Irish farms: predominant mould and yeast species and features of bales and silage. Grass Forage Sci 63: 121–137.

[mbt213552-bib-0042] Pahlow, G. , Muck, R.E. , Driehuis, F. , Elferink, S. , and Spoelstra, S.F. (2003) Microbiology of ensiling. J Agronomy 42: 31–94.

[mbt213552-bib-0043] Reboux, G. , Reiman, M. , Roussel, S. , Taattola, K. , Millon, L. , Dalphin, J. , and Piarroux, R. (2006) Impact of agricultural practices on microbiology of hay, silage and flour on Finnish and French farms. Ann Agric Environ Med 13: 267–273. 17196000

[mbt213552-bib-0044] Richard, E. , Heutte, N. , Bouchart, V. , and Garon, D. (2009) Evaluation of fungal contamination and mycotoxin production in maize silage. Anim Feed Sci Tech 148: 309–320.

[mbt213552-bib-0045] Rossi, F. , and Dellaglio, F. (2007) Quality of silages from Italian farms as attested by number and identity of microbial indicators. J Appl Microbiol 103: 1707–1715. 17953581 10.1111/j.1365-2672.2007.03416.x

[mbt213552-bib-0046] Rousk, J. , and Bååth, E. (2007) Fungal biomass production and turnover in soil estimated using the acetate‐in‐ergosterol technique. Soil Biol Biochem 39: 2173–2177.

[mbt213552-bib-0047] Seyedmousavi, S. , Bosco, S.D.M. , De Hoog, S. , Ebel, F. , Elad, D. , Gomes, R.R. , *et al* (2018) Fungal infections in animals: a patchwork of different situations. Med Mycol 56: S165–S187. 10.1093/mmy/myx104 PMC6251577 29538732

[mbt213552-bib-0048] Sharpe, R.A. , Le Cocq, K. , Nikolaou, V. , Osborne, N.J. , and Thornton, C.R. (2016) Identifying risk factors for exposure to culturable allergenic moulds in energy efficient homes by using highly specific monoclonal antibodies. Environ Res 144: 32–42. 26546982 10.1016/j.envres.2015.10.029

[mbt213552-bib-0049] Tangni, E.K. , Pussemier, L. , Bastiaanse, H. , Haesaert, G. , Foucart, G. , and Van Hove, F. (2013) Presence of mycophenolic acid, roquefortine C, citrinin and ochratoxin A in maize and grass silages supplied to dairy cattle in Belgium. J Anim Sci Adv 3: 598–612.

[mbt213552-bib-0050] Thornton, C.R. (2005) Use of monoclonal antibodies to quantify the dynamics of alpha‐galactosidase and endo‐1,4‐beta‐glucanase production by *Trichoderma hamatum* during saprotrophic growth and sporulation in peat. Environ Microbiol 7: 737–749. 15819855 10.1111/j.1462-2920.2005.00747.x

[mbt213552-bib-0051] Thornton, C.R. (2008a) Development of an immunochromatographic lateral‐flow device for rapid serodiagnosis of invasive aspergillosis. Clin Vaccine Immunol 15: 1095–1105. 18463222 10.1128/CVI.00068-08 PMC2446636

[mbt213552-bib-0052] Thornton, C.R. (2008b) Tracking fungi in soil with monoclonal antibodies. Eur J Plant Pathol 121: 347–353.

[mbt213552-bib-0053] Thornton, C.R. , Dewey, F.M. , and Gilligan, C.A. (1994) Development of a monoclonal antibody‐based enzyme‐linked‐immunosorbent‐assay for the detection of live propagules of trichoderma‐harzianum in a peat‐bran medium. Soil Biol Biochem 26: 909–920.

[mbt213552-bib-0054] Thornton, C. , Johnson, G. , and Agrawal, S. (2012) Detection of invasive pulmonary aspergillosis in haematological malignancy patients by using lateral‐flow technology. J Vis Exp 61: e3721. 10.3791/3721 PMC3460586 22473419

[mbt213552-bib-0055] Tothill, I.E. , Harris, D. , and Magan, N. (1992) The relationship between fungal growth and ergosterol content of wheat grain. Mycol Res 96: 965–970.

[mbt213552-bib-0056] VSN International (2015) Genstat for Windows 18th Edition. Hemel Hempstead, UK: VSN International URL http://Genstat.co.uk.

[mbt213552-bib-0057] Waldmann, T.A. (1991) Monoclonal antibodies in diagnosis and therapy. Science 252: 1657–1662. 2047874 10.1126/science.2047874

[mbt213552-bib-0058] Wilkinson, J. , and Davies, D. (2013) The aerobic stability of silage: key findings and recent developments. Grass Forage Sci 68: 1–19.

[mbt213552-bib-0059] Wilkinson, J.M. , and Toivonen, M.I. (2003) World silage: a survey of forage conservation around the world. Marlow, UK: Chalcombe Publ.

[mbt213552-bib-0060] Williams, S.D. , and Shinners, K.J. (2012) Farm‐scale anaerobic storage and aerobic stability of high dry matter sorghum as a biomass feedstock. Biomass Bioenergy 46: 309–316.

[mbt213552-bib-0061] Williams, A. , Critten, D. , and Reynolds, A. (1995) A mathematical model of the aerobic deterioration of silage. Grass Forage Sci 50: 132–146.

[mbt213552-bib-0062] Woolford, M.K. (1990) The detrimental effects of air on silage. J Appl Microbiol 68: 101–116. 10.1111/j.1365-2672.1990.tb02554.x 2180886

[mbt213552-bib-0063] Zhao, X. , Lin, Q. , and Brookes, P. (2005) Does soil ergosterol concentration provide a reliable estimate of soil fungal biomass? Soil Biol Biochem 37: 311–317.

[mbt213552-bib-0064] Zhao, F. , Li, R. , Xiao, S. , Diao, H. , Viveiros, M.M. , Song, X. , and Ye, X. (2013) Postweaning exposure to dietary zearalenone, a mycotoxin, promotes premature onset of puberty and disrupts early pregnancy events in female mice. Toxicol Sci 132: 431–442. 23291560 10.1093/toxsci/kfs343 PMC3595522

[mbt213552-bib-0065] Zhu, W.Y. , Theodorou, M.K. , Longland, A.C. , Nielsen, B.B. , Dijkstra, J. , and Trinci, A.P. (1996) Growth and survival of anaerobic fungi in batch and continuous‐flow cultures. Anaerobe 2: 29–37.

