# Supplementary material for: Application of monoclonal antibodies in quantifying fungal growth dynamics during aerobic spoilage of silage
Source: Microb Biotechnol. 2020 Mar 10;13(4):1054–65. doi: 10.1111/1751-7915.13552 (PMC7264882; doi:10.1111/1751-7915.13552)
Supplement: Supplementary file 1 — Table S1. Summary information for each antibody used in the study. [file MBT2-13-1054-s001.docx]

**Supplementary Table 1**. Summary information for each antibody used in the study.

| **Monoclonal antibody clone name** | **Genera detected** | **Target epitope/ antigen** | **Isotype** | **Reference** |
| --- | --- | --- | --- | --- |
| MF2 | All *Trichoderma* species | 35-200kDa glycoprotein | IgM | (Thornton *et al.*, 2002) |
| JF5 | *Aspergillus* and *Penicillium* species | 50 to >200kDa mannoprotein | IgG3 | (Rolle *et al.*, 2016) |
| IE3 | Pan-fungal for filamentous ascomycetes | 25 to >250kDa glycoprotein | IgM |  |
| MC3 | *Candida albicans, C. auris, C. dubliniensis, C. guilliermondii, C. lusitaniae, C. tropicalis, C. famata, C. pseudotropicalis, C. palmioleophila* | Putative b-1,2-mannan epitope in C. albicans blastospore, hyphal and pseudo-hyphal mannoproteins and phospholipomannans | IgG3 | (Morad *et al.*, 2018) |
| ED7 | All Fusarium species | 200kDa glycoprotein | IgM | (Al‐Maqtoofi and Thornton, 2016) |

Al‐Maqtoofi, M., and Thornton, C.R. (2016) Detection of human pathogenic *Fusarium* species in hospital and communal sink biofilms by using a highly specific monoclonal antibody, *Environ microbiol* **18**: 3620-3634.

Morad, H.O., Wild, A.-M., Wiehr, S., Davies, G., Maurer, A., Pichler, B.J., and Thornton, C.R. (2018) Pre-clinical Imaging of Invasive Candidiasis Using ImmunoPET/MR, *Front microbiol* **9**: 1996.

Rolle, A.-M., Hasenberg, M., Thornton, C.R., Solouk-Saran, D., Männ, L., Weski, J., et al. (2016) ImmunoPET/MR imaging allows specific detection of *Aspergillus fumigatus* lung infection in vivo,  *Proc Natl Acad Sci U S A* **113**: E1026-E1033.

Thornton, C.R., Pitt, D., Wakley, G.E., and Talbot, N.J. (2002) Production of a monoclonal antibody specific to the genus *Trichoderma* and closely related fungi, and its use to detect *Trichoderma* spp. in naturally infested composts, *Microbiology-Sgm* **148**: 1263-1279.
